# Correction: Therapeutic interventions on human breast cancer xenografts promote systemic dissemination of oncogenes

**DOI:** 10.1371/journal.pone.0301650

**Published:** 2024-03-28

**Authors:** Gorantla V. Raghuram, Kavita Pal, Gaurav Sriram, Afzal Khan, Ruchi Joshi, Vishalkumar Jadhav, Sushma Shinde, Alfina Shaikh, Bhagyeshri Rane, Harshada Kangne, Indraneel Mittra

The legends for S4 Fig are missing from the article. Please view the correct S4 Fig and captions below.

## Supporting information

S4 Fig **Figure showing fluorescently dual-labelled cfChPs in mouse brain (a and b):** Fluorescently dually labelled MDA-MB-231 cells when injected intravenously into SCID mice die upon reaching the brain and release cfChPs which accumulate in their brain cells. **a.** Representative image of dually fluorescently labelled MDA-MB-231 cells. **b.** Fluorescent microscopy image of brains of mice injected with dually labelled MDA-MB-231 cells to demonstrate that the cells die upon reaching the brain to release dually labelled fluorescent particles representing cfChPs which accumulate in their brain cells.
(TIF)
