# Supplementary figures and images for: Therapeutic interventions on human breast cancer xenografts promote systemic dissemination of oncogenes
Source: PLoS One. 2024 Feb 12;19(2):e0298042. doi: 10.1371/journal.pone.0298042 (PMC10861051; doi:10.1371/journal.pone.0298042)

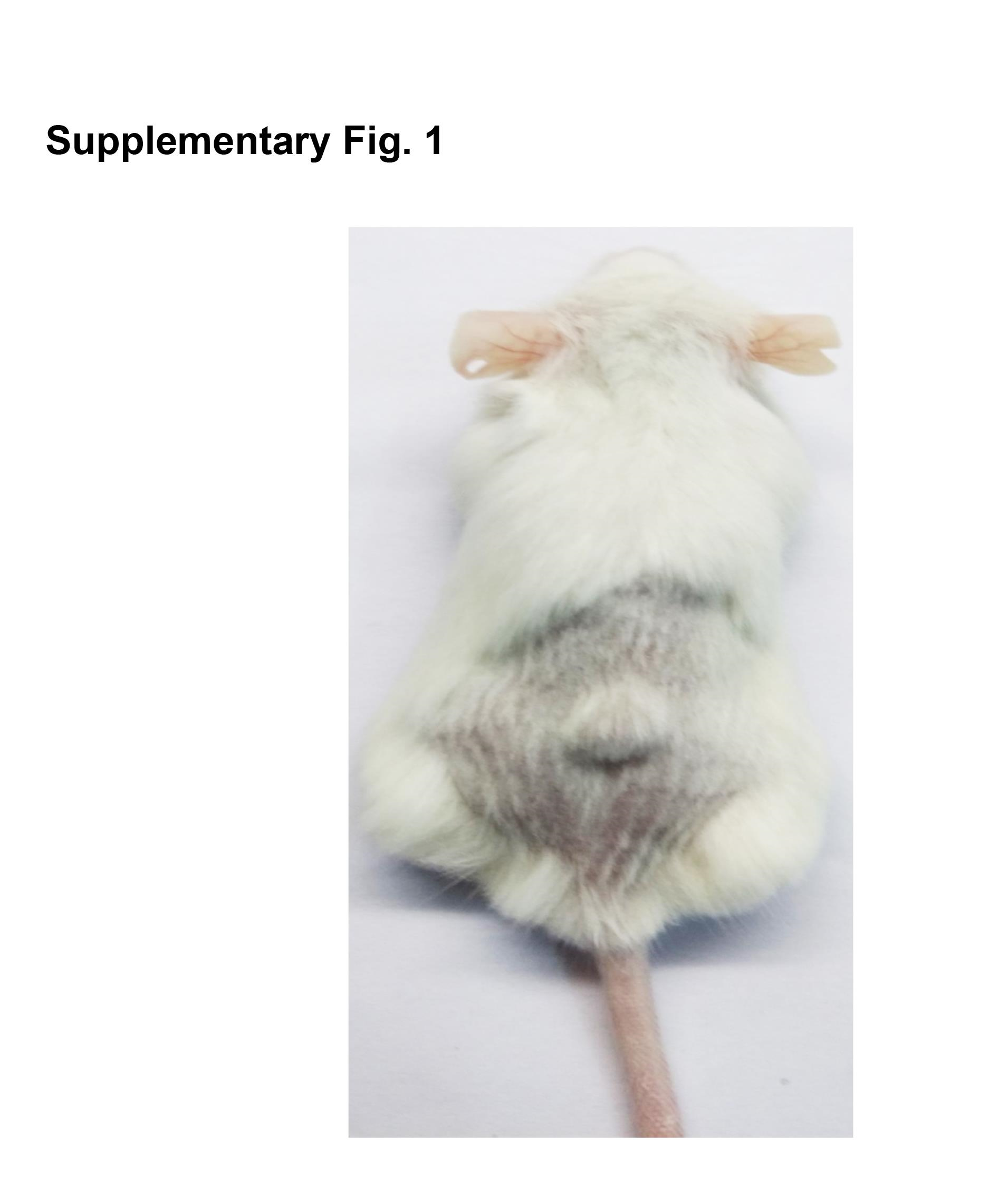

Supplement: S1 Fig — (TIF) [file pone.0298042.s001.tif]

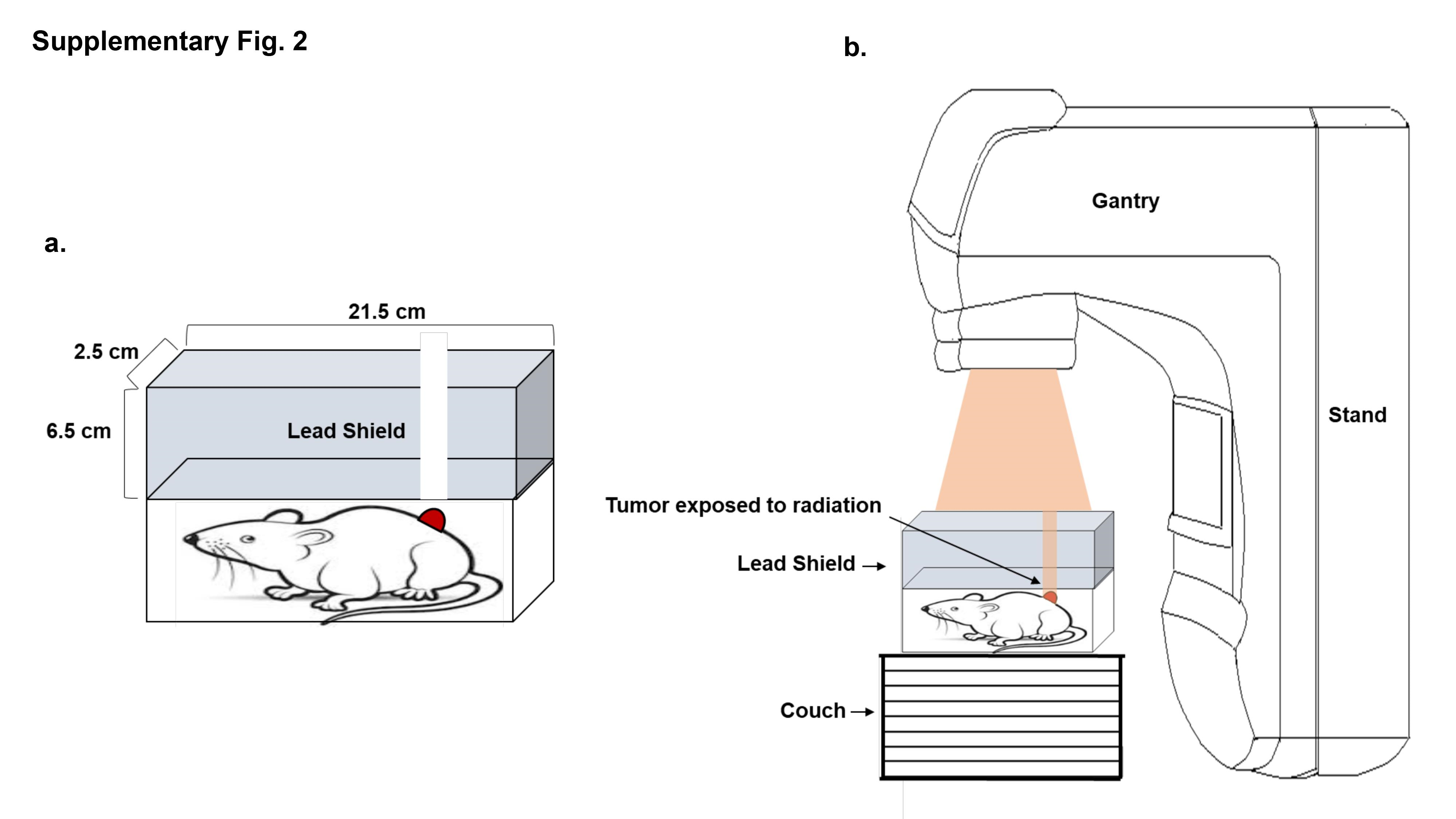

Supplement: S2 Fig — (TIF) [file pone.0298042.s002.tif]

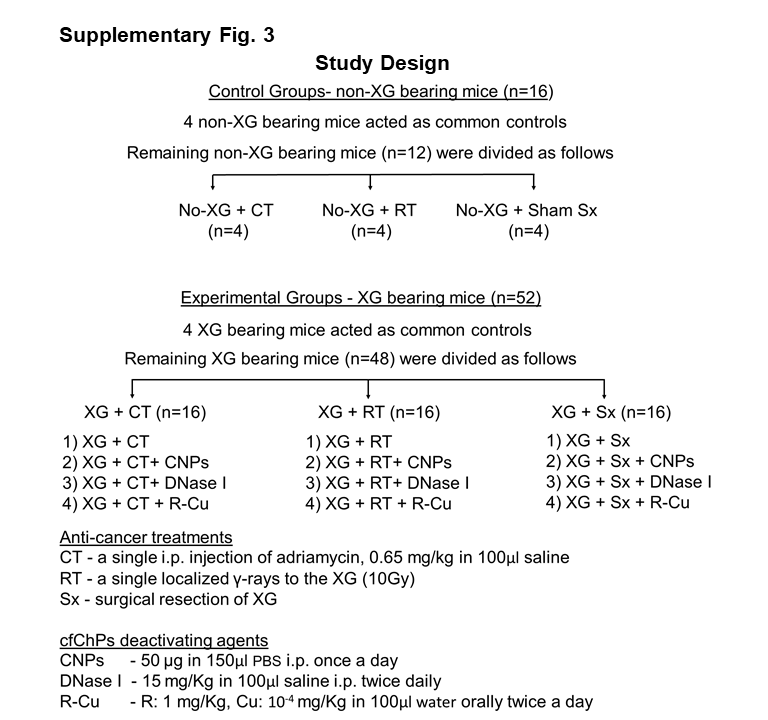

Supplement: S3 Fig — (TIF) [file pone.0298042.s003.tif]

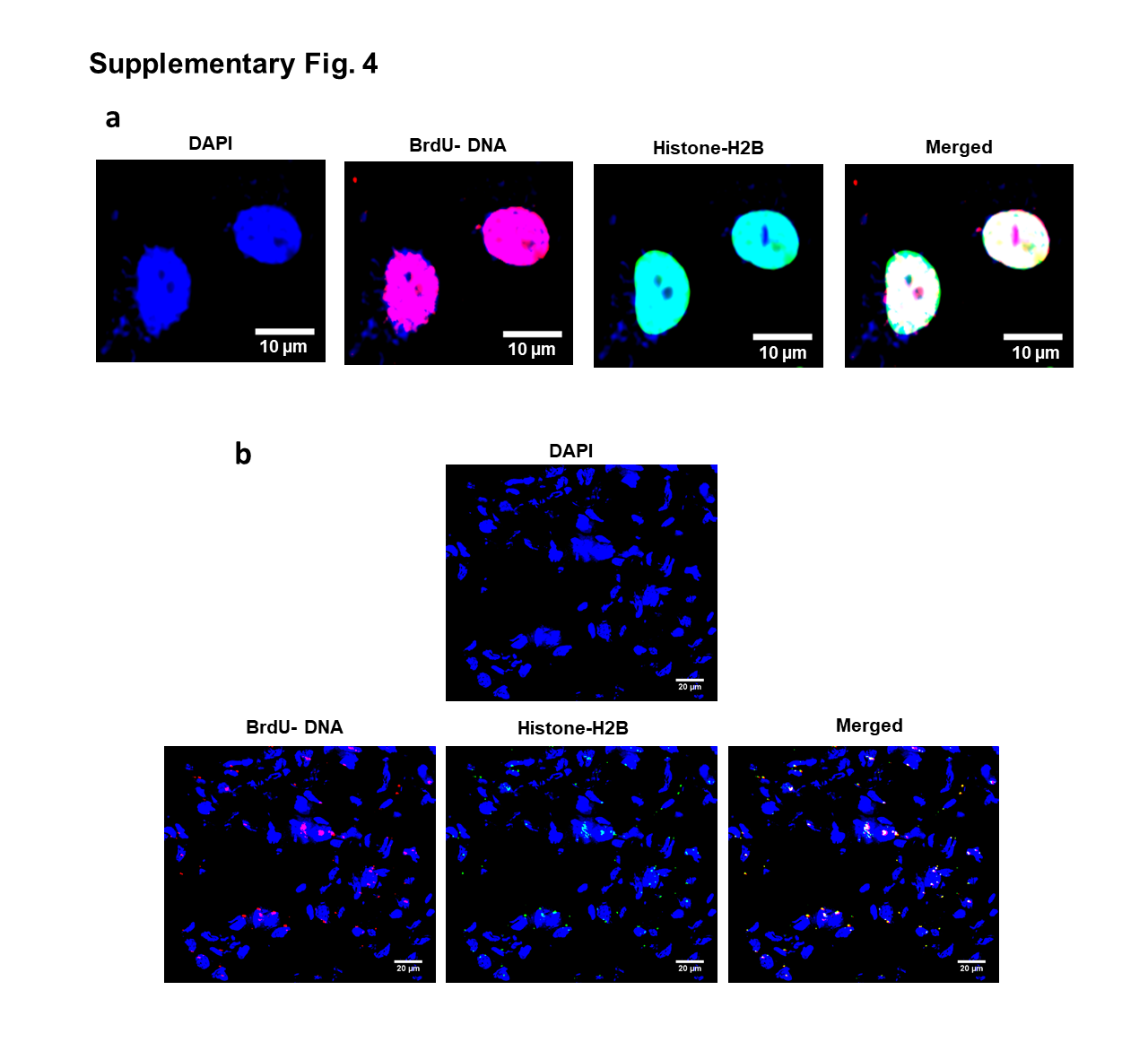

Supplement: S4 Fig — (TIF) [file pone.0298042.s004.tif]
