# Supplementary material for: Therapeutic interventions on human breast cancer xenografts promote systemic dissemination of oncogenes
Source: PLoS One. 2024 Feb 12;19(2):e0298042. doi: 10.1371/journal.pone.0298042 (PMC10861051; doi:10.1371/journal.pone.0298042)
Supplement: S1 Table — (DOCX) [file pone.0298042.s005.docx]

**Supplementary Table 1:**

**Source of FISH probes and antibodies.**

**FISH Probe**

| **Sr. No.** | **FISH Probe** | **Catalogue No.** | **Company / Vendor** |
| --- | --- | --- | --- |
|  | Human Genomic DNA (probe) Red | Custom synthesized | Applied Spectral Imaging, Israel |

**Primary antibodies**

| **Sr. No.** | **Primary antibody** | **Catalogue No.** | **Company / Vendor** |
| --- | --- | --- | --- |
|  | HLA-ABC1 | ab70328 | Abcam, UK |
|  | c-MYC | M4439 | Sigma-Merck-Millipore, Germany |
|  | c-Raf | MA5-41270 | Invitrogen- Thermo Fisher Scientific, USA |
|  | p-EGFR | 4404S | Cell Signaling Technologies,USA |
|  | HRAS | Orb38488 | Biorbyt Biotech, USA |
|  | p-AKT | 4060S | Cell Signaling Technologies,USA |
|  | FGFR 3 | 4574S | Cell Signaling Technologies,USA |
|  | PDGFRA | HPA004947 | Atlas Antibodies,USA |
|  | c-abl Antibody | PA514762 | Invitrogen- Thermo Fisher scientific, USA |

All antibodies were specific to humans except that for p-AKT which was common for human and mouse.

| **Sr. No.** | **Secondary antibody** | **Catalogue No.** | **Company / Vendor** |
| --- | --- | --- | --- |
|  | Goat Anti-Rabbit IgG (H+L) FITC Conjugate Secondary Antibody | AP307F | Merck-Millipore Sigma, Germany |
|  | Goat Anti-Mouse IgG H&L (FITC) Pre-Adsorbed Secondary Antibody | ab7064 | Abcam, Cambridge, UK |

**Secondary antibodies**
