# Supplementary material for: Therapeutic interventions on human breast cancer xenografts promote systemic dissemination of oncogenes
Source: PLoS One. 2024 Feb 12;19(2):e0298042. doi: 10.1371/journal.pone.0298042 (PMC10861051; doi:10.1371/journal.pone.0298042)

**Supplementary Table 2.**

**The ARRIVE Essential 10**

| **1.** | **Study design** | 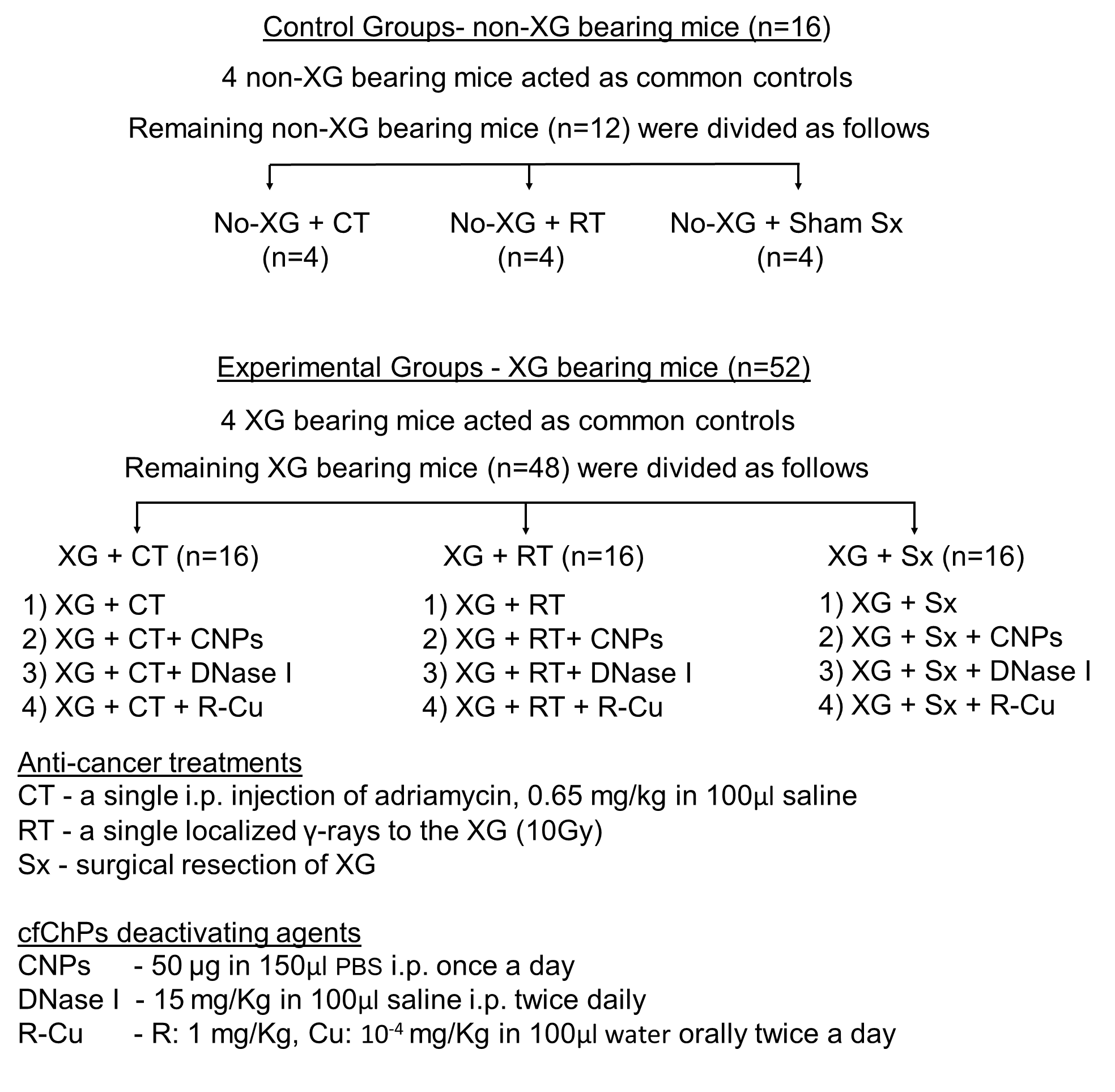 |
| --- | --- | --- |
| **2.** | **Sample size** | Total sample size was 68 SCID mice. Control group had 16 mice and Experimental group had 52. |
| **3.** | **Inclusion and exclusion criteria** | Inbred female NOD SCID (NOD.Cg-Prkdcscid/J) mice of age 6-8 weeks and weight range of 20-22 g. Those mice in the age range but not in the weight range were excluded from the study. |
| **4.** | **Randomisation** | Mice were randomly grouped during initiation of the study**.** After inoculation of MDA MB cells, mice were observed for tumour formation. Once the tumour volume reached to 0.125mm^3^**,** mice were divided in to groups. |
| **5.** | **Blinding** | NA, since there are different treatments given to mice, it was not possible to blind during experimental procedure. |
| **6.** | **Outcome measures** | Experiment no.1:-  Involve XG bearing mice without anti-cancer treatments. Their brain sections were analyzed for detection of human DNA and human onco-proteins by immuno-FISH.  Experiment no.2:-  Involve XG bearing mice receiving anti-cancer treatments. Statistical comparison was performed on the effects of anti-cancer treatments, with and without cfChPs deactivating agents, on human DNA and c-Myc onco-protein signals in brains cells by immuno-FISH. |
| **7.** | **Statistical methods** | Statistical comparison between the xenograft bearing group and the two control groups, and that between the xenograft bearing group and the three anti-cancer treatment groups (CT, RT and Sx) was done by two-tailed student t-test using GraphPad Prism 6.0 (https://www.graphpad.com/, GraphPad Software, Inc., USA). Statistical comparison between the three treatment groups (CT, RT and Sx) and those additionally treated with the three cfChPs deactivating agents was performed by One-way ANOVA using the same software. |
| **8.** | **Experimental animals** | Inbred female NOD SCID (NOD.Cg-Prkdcscid/J) mice were used in this study. |
| **9.** | **Experimental procedures** | **Creation of xenografts**  NOD SCID mice (6-8 week old) were inoculated under the lower dorsal skin with 1 X 10^6^ MDA-MB-213 human breast cancer cells and xenografts were allowed to grow to a size of ~0.125 cm^3^, which usually took ~6 weeks, when the experiments were initiated (Supplementary Fig. 1).  **Therapeutic interventions:**  Chemotherapy: Mice were administered a single i.p. injection of Adriamycin, 0.65 mg/kg in 100µl of saline  Tumour irradiation: Mice were anesthetized using ketamine (80 mg/kg IP) and xylazine (10 mg/kg IP) and placed in a polypropylene box. The box was placed on the couch of a telecobalt machine (Bhabatron-II)^©^ and positioned in such a way that the apex of the tumor was at the center of the aperture of the machine. The rest of the body of the mice was shielded from radiation using 6.5 cm-thick lead shields (Supplementary Fig. 2).A dose of radiation was delivered to the tumors (10Gy).  Surgical excision: Mice were anesthetized using ketamine (80 mg/kg IP) and xylazine (10 mg/kg IP) and the xenografts were surgically excised.  **cfChPs deactivating agents:**  The anti-cancer therapies were given either alone or in conjunction with three different cfChPs deactivating agents viz. anti-histone antibody complexed nanoparticles (CNPs) [12], DNase I or a combination of the nutraceuticals Resveratrol and copper (R-Cu) [13,14]. Combining Resveratrol (R) with copper (Cu) leads to the generation of free radicals [15] which can effectively deactivate cfChPs by degrading their DNA components [13,14].  Anti-histone antibody complexed nanoparticles (CNPs): CNPs were prepared according to the method reported by us earlier [12] except that histone H4 IgG was exclusively used for preparing CNPs. CNPs, 50 μg in 150 μl PBS, were administered once a day for 5 days.  DNase I: DNase I (Sigma-Aldrich; Catalogue No- DN25-1G) dissolved in saline was administered twice daily at a dose of 15 mg/kg in 100 µl of saline i.p.  Resveratrol-copper (R-Cu): R: 1 mg/Kg in 100µl water, and Cu: 10^-4^ mg/Kg in 100µl water, were administered by oral gavage one after the other twice daily. Resveratrol (Trade name—TransMaxTR) was sourced from Biotivia LLC, USA; Copper (Trade name—Chelated Copper) was sourced from J.R. Carlson Laboratories Inc. USA. Mice were sacrificed under CO2 atmosphere by cervical dislocation under supervision of FELASA trained animal facility personnel 5 days (i.e. on day 6) after starting anti-cancer treatments; cfChPs deactivating agents were started 4h prior to commensing anti-cancer therapies. Brains of mice were removed, fixed in formalin and FFPE sections were prepared for analysis.  The FFPE sections of the brain were analyzed by immuno-FISH using a human specific whole genomic probe and specific antibodies against human HLA-ABC antigen and eight different human oncogenes viz. c-Myc; c-Raf, p-EGFR, HRAS, p-AKT, FGFR 3, PDGFRA and c-Abl. For FISH analysis, 500 cells were examined for detection of human DNA signals (at a magnification of X60) and percentage of cells showing positive signals was calculated. For onco-protein analysis, 1000 cells were examined for detection of human c-Myc signals (at a magnification of X40) and percentage of cells showing positive signals for human c-Myc protein was calculated. Details of the FISH probe and various antibodies used in this study are provided in the Supplementary Table 1. |
| **10.** | **Results** | Using immuno-FISH analysis on brain cells of xenograft bearing mice, we detected multiple co-localizing signals of human DNA and HLA-ABC in their brain cells (Fig.1a). Since the HLA-ABC antigen is unique to humans, and does not exist in mice, this finding provided strong support for the conclusion that DNA fragments carrying the HLA-ABC gene had been released from the dying human xenograft cells and had migrated to mouse brain cells via circulation. We also detected co-localizing signals of human DNA and eight human onco-proteins that we examined in brain cells of mice. The onco-proteins included c-Myc; c-Raf, p-EGFR, HRAS, p-AKT, FGFR 3, PDGFRA and c-Abl (Fig. 1b). This finding indicated that the respective oncogenes had also been released from dying xenograft cells and carried via the blood stream to brain cells wherein they had expressed their respective proteins.  We next undertook a quantitative comparative analysis of human DNA and c-Myc onco-protein signals in brain cells following three anti-cancer interventions viz. a single intraperitoneal injection of Adriamycin, a single shot of localized radiation or surgical excision of the xenograft. These therapeutic interventions were given either alone or concurrently with three different cfChPs deactivating agents, namely, anti-histone antibody complexed nanoparticles (CNPs), DNase 1 and a combination of Resveratrol and copper (Fig. 2, a and b). The analysis was done in a blinded fashion such that the examiner was unaware of the group to which the immune-FISH slides belonged. As expected, no human DNA or c-Myc signals were detected in brain cells of mice in the two control groups viz. control mice without xenograft and control mice without xenograft but receiving anti-cancer interventions (Fig. 2, a and b). However, xenograft bearing mice (without therapeutic interventions) showed that a significant number of brain cell nuclei harbored human DNA (13.73%) and c-Myc onco-protein (3.70%) signals. This data indicated that dying cells of the xenografts had released their DNA carrying the c-Myc oncogene into the circulation which had migrated to the brain during the xenograft’s growth span of ~6 weeks. The number of human DNA and c-Myc signals increased markedly following the three therapeutic interventions. With respect to DNA, the maximum increase in signals was seen with CT (13.73% vs 26.33%, p < 0.001), followed by RT (13.73% vs 21.83%, p < 0.01), followed by Sx (13.73% vs 15.83%, p < 0.05). With respect to c-Myc, the number of signals for CT were 3.70% vs 8.08% (p < 0.01) and for RT were 3.70% vs 9.33% (p < 0.001). However, for Sx, the difference in number of signals in untreated and treated groups was not statistically significant (3.70% vs 3.37%). Concurrent treatment of mice with all three cfChPs deactivating agents showed remarkable and statistically significant reduction in both human DNA and c-Myc signals with p values ranging between < 0.05 and < 0.01 (Fig. 2, a and b). |

**The Recommended Set**

| **1.** | **Abstract** | Metastatic dissemination following successful treatment of the primary tumour remains a common cause of death. There is mounting evidence that therapeutic interventions themselves may promote development of metastatic disease. Based on our earlier finding that cell-free chromatin particles (cfChPs) released from dying cancer cells are potentially oncogenic, we hypothesized that therapeutic interventions may disseminate the disease via medium of cfChPs released from therapy induced dying cancer cells. To test this hypothesis, we generated xenografts of MDA-MB-231 human breast cancer cells in mice, and using immuno-fluorescence and FISH analysis, detected multiple co-localizing signals of human DNA and eight human onco-proteins in the mouse brain cells. Number of signals increased dramatically following treatment with chemotherapy, localized radiotherapy or surgery, which could be minimized by concurrent treatment with three different cfChPs deactivating agents. These results suggest that therapeutic interventions may potentially encourage metastatic spread of cancer which is preventable by deactivating cell-free chromatin. |
| --- | --- | --- |
| **2.** | **Background** | Systemic dissemination following successful treatment of the primary tumour remains a common cause of death from the disease. There is mounting evidence that therapeutic interventions themselves may promote metastatic spread of cancer [1]. This possibility has been raised with respect to all three modalities of cancer treatment viz. chemotherapy [2,3], radiotherapy [4,5] and surgery [6,7]. Based on our earlier finding that cell-free chromatin particles (cfChPs) that are released from dying cancer cells are potentially oncogenic [8], we hypothesized that therapeutic interventions may disseminate the disease via release of cfChPs from therapy induced dying cancer cells. In keeping with the classical report of Isaiah Fidler [9], we had also observed that vast majority of cancer cells intravenously injected into mice had died upon reaching target organs to release cfChPs which integrated into the genomes of target cells. This resulted in dsDNA breaks marked by phosphorylation of H2AX, and activation of inflammatory cytokines NFκB, IL-6, TNFα and IFNγ. Since concurrent activation of DNA damage and inflammation are potent stimuli of oncogenic transformation [10,11], we had hypothesized that cfChPs released from therapy induced dying cancer cells are the critical agents that induce metastatic dissemination of cancer [8]. In the present study, we tested this hypothesis in a pre-clinical model using human breast cancer xenografts in SCID mice and found that therapeutic interventions viz. chemotherapy, radiotherapy and surgical excision of the xenograft leads to systemic disseminates of cfChPs carrying human oncogenes which integrate into brain cells of mice. |
| **3.** | **Objectives** | To study whether therapeutic interventions on human cancer xenografts and systemic dissemination of oncogenes |
| **4.** | **Ethical statement** | The experimental protocol of this study was approved by the Institutional Animal Ethics Committee (IAEC) of the Advanced Centre for Treatment, Research and Education in Cancer (ACTREC), Tata Memorial Centre (TMC), Navi Mumbai, India under permission number 32/2016. The experiments were carried out in compliance with the ethical regulations and humane endpoint criteria of IAEC and ARRIVE guidelines. The Plos One checklist of ARRIVE guidelines is given in Supplementary table. 2. |
| **5.** | **Housing and husbandry** | Inbred female NOD SCID (NOD.Cg-Prkdcscid/J) mice were used in this study. They were obtained from the institutional animal facility and were maintained according to IAEC standards. They were housed under maximum-barrier facilities in sterilized cages with filter tops and sterile bedding material. The mice were fed γ-irradiated sterilized food and water ad libitum and kept under 12-h light/dark cycle with free access to water and food. A HVAC system was used to provide controlled room temperature, humidity and air pressure.  ACTREC- IAEC maintains respectful treatment and care of animals in scientific research. It aims that the use of animals in research contributes to the advancement of knowledge following ethical and scientific necessities. All scientists and technicians involved in this study have undergone training in ethical handling and management of animals under supervision of FELASA certified attending veterinarians. They affirm that respect for all forms of life is an inherent characteristic of biological and medical scientists who conduct research involving animals. Animals were euthanized under sterile conditions at appropriate time points under CO_2_ atmosphere by cervical dislocation under supervision of FELASA trained animal facility personnel. |
| **6.** | **Animal care and monitoring** | All welfare considerations were taken to minimize any suffering and distress due to creation of the tumour xenografts. While considering humane end points, it was ensured that xenografts did not reach a size of 10 mm in any one dimension. In addition, activity and weight loss of more than 15 % were considered as humane end points. Mice were observed for the above humane end points once every two days. None of the animals were found to have reached the above humane end points at the time of sacrifice. None of the animals died during the total duration of experiment of ~48 days.  The PLOS ONE Humane Endpoints Checklist is given as Supplementary Table. 3. |
| **7.** | **Interpretation/ scientific implications** | Using immuno-FISH analysis on brain cells of xenograft bearing mice, we detected multiple co-localizing signals of human DNA and HLA-ABC in their brain cells (Fig.1a). Since the HLA-ABC antigen is unique to humans, and does not exist in mice, this finding provided strong support for the conclusion that DNA fragments carrying the HLA-ABC gene had been released from the dying human xenograft cells and had migrated to mouse brain cells via circulation. We also detected co-localizing signals of human DNA and eight human onco-proteins that we examined in brain cells of mice. The onco-proteins included c-Myc; c-Raf, p-EGFR, HRAS, p-AKT, FGFR 3, PDGFRA and c-Abl (Fig. 1b). This finding indicated that the respective oncogenes had also been released from dying xenograft cells and carried via the blood stream to brain cells wherein they had expressed their respective proteins.  We next undertook a quantitative comparative analysis of human DNA and c-Myc onco-protein signals in brain cells following three anti-cancer interventions viz. a single intraperitoneal injection of Adriamycin, a single shot of localized radiation or surgical excision of the xenograft. These therapeutic interventions were given either alone or concurrently with three different cfChPs deactivating agents, namely, anti-histone antibody complexed nanoparticles (CNPs), DNase 1 and a combination of Resveratrol and copper (Fig. 2, a and b). The analysis was done in a blinded fashion such that the examiner was unaware of the group to which the immune-FISH slides belonged. As expected, no human DNA or c-Myc signals were detected in brain cells of mice in the two control groups viz. control mice without xenograft and control mice without xenograft but receiving anti-cancer interventions (Fig. 2, a and b). However, xenograft bearing mice (without therapeutic interventions) showed that a significant number of brain cell nuclei harbored human DNA (13.73%) and c-Myc onco-protein (3.70%) signals. This data indicated that dying cells of the xenografts had released their DNA carrying the c-Myc oncogene into the circulation which had migrated to the brain during the xenograft’s growth span of ~6 weeks. The number of human DNA and c-Myc signals increased markedly following the three therapeutic interventions. With respect to DNA, the maximum increase in signals was seen with CT (13.73% vs 26.33%, p < 0.001), followed by RT (13.73% vs 21.83%, p < 0.01), followed by Sx (13.73% vs 15.83%, p < 0.05). With respect to c-Myc, the number of signals for CT were 3.70% vs 8.08% (p < 0.01) and for RT were 3.70% vs 9.33% (p < 0.001). However, for Sx, the difference in number of signals in untreated and treated groups was not statistically significant (3.70% vs 3.37%). Concurrent treatment of mice with all three cfChPs deactivating agents showed remarkable and statistically significant reduction in both human DNA and c-Myc signals with p values ranging between < 0.05 and < 0.01 (Fig. 2, a and b). |
| **8.** | **Generalisability/ translation** | Although systemic dissemination following successful treatment of the primary tumour is a common cause of death from the disease, an explanation for this paradox has remained elusive. We have shown here that DNA fragments released from therapy induced dying cancer cells, and carrying oncogenes with them, can enter into the systemic circulation and be carried to distant organs, such as the brain, with the potential to oncogenically transform their constituent cells. The fact that CNPs, which deactivate cfChPs by binding to their histone components [12], could effectively prevent c-Myc dissemination indicates that the agents that carried the c-Myc oncogene to brain cells were in fact cell-free chromatin particles released from dying xenograft cells. This finding is consistent with our hypothesis that cell-free chromatin particles are the critical oncogenic agents responsible for inducing metastasis [8].  Whether therapy induced dissemination of oncogenes to brain cells would lead to development of metastases was not investigated in our short-term study. Nonetheless, the fact that cfChPs have the ability to concurrently activate two critical hallmarks of cancer viz. genomic instability and inflammation [8] suggests that prolonged observation is called for. Future long-term experiments should also explore whether cfChPs deactivating agents given concurrently with anti-cancer treatments would prevent metastatic spread. These agents would have the added advantage, when used as adjuncts to cancer treatment, of preventing toxic side effects of chemotherapy [16,17] and radiotherapy [18]. |
| **9.** | **Protocol registration** | The experimental protocol of this study was approved by the Institutional Animal Ethics Committee (IAEC) of the Advanced Centre for Treatment, Research and Education in Cancer (ACTREC), Tata Memorial Centre (TMC), Navi Mumbai, India under permission number 32/2016. |
| **10.** | **Data access** | All data are contained within the manuscript. Any additional data will be provided on reasonable request. |
| **11.** | **Declaration of interests** | The authors declare no competing interests. |


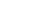


AR


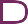

Supplement: S2 Table — (DOCX) [file pone.0298042.s006.docx]
