# Supplementary material for: Therapeutic interventions on human breast cancer xenografts promote systemic dissemination of oncogenes
Source: PLoS One. 2024 Feb 12;19(2):e0298042. doi: 10.1371/journal.pone.0298042 (PMC10861051; doi:10.1371/journal.pone.0298042)
Supplement: S3 Table — (DOCX) [file pone.0298042.s007.docx]

**Supplementary Table 3**

*PLOS ONE* Humane Endpoints Checklist

***PLOS ONE* manuscript number:** **PONE-D-23-14930**

**Humane endpoints used for all animals involved in the study**

|  | **Recommendation** | **Section/Paragraph** |
| --- | --- | --- |
| **If humane endpoints* were used, report the following:** | | |
| **1** | **The specific criteria used to determine when animals should be euthanized** | **Any one dimension if more than 10 mm, loss of weight or activity by 15 % or more.** |
| **2** | **Once animals reached endpoint criteria, the amount of time elapsed before euthanasia** | **None of the animals reach the humane endpoints during the experimental tenure of ~48 days** |
| **3** | **Whether any animals died before meeting criteria for euthanasia** | **No** |
| **If humane endpoints* were not used, report the following:** | | |
| **1** | **A scientific and ethical justification for the study design, including the reasons why humane endpoints could not be used, and discussion of alternatives that were considered but could not be used** | **NA** |
| **2** | **Whether the institutional animal ethics committee specifically reviewed and approved the anticipated mortality in the study design** | **NA** |

**ITEM 2.** **Include the following details of the study design and outcomes.**

|  | **Recommendation** | **Section/Paragraph** |
| --- | --- | --- |
| **1** | **The duration of the experiment** | **48-50 days** |
| **2** | **The numbers of animals used, euthanized, and found dead (if any); the cause of death for all animals** | **68** |
| **3** | **How frequently animal health and behavior were monitored** | **Once in every two days** |
| **4** | **All animal welfare considerations taken, including efforts to minimize suffering and distress, use of analgesics or anaesthetics, or special housing conditions** | **All welfare considerations were taken to minimize any suffering and distress due to creation of the tumour xenografts such as proper handling of mice. Tumours were induced at the lower flank so that the movement of mice is maintained at normal. We did not find any requirement of giving analgesia. However, euthanasia was performed under CO2 atmosphere under the guidance of FELASA trained veterinarian.** |
| **5** | **Any special training in animal care or handling provided for research staff** | **All scientists and technicians involved in this study have undergone training in ethical handling and management of animals under supervision of FELASA certified attending veterinarians.** |
