# Supplementary material for: Therapeutic interventions on human breast cancer xenografts promote systemic dissemination of oncogenes
Source: PLoS One. 2024 Feb 12;19(2):e0298042. doi: 10.1371/journal.pone.0298042 (PMC10861051; doi:10.1371/journal.pone.0298042)
Supplement: Attachment — Submitted filename: revision 2.pdf [file pone.0298042.s009.pdf]

Comments on the revised manuscript

1. Supplementary figure.3 has to be replaced by a more high resolution image.

2. Point no.5 from the previous review (reviewer 2) has not been addressed properly. In the manuscript, the authors have shown increased c-Myc signal in the brain of tumor bearing animals with/without therapeutic intervention at varying intensity compared to control group where there is no signal. However, the antibody used here is reactive to mouse c-Myc and human c-Myc. Hence, the question of specificity still remains. Moreover, the possibility that the internalized DNA could be activating pathways like cGAS-STING cannot be ruled out. These pathways could be responsible for the elevated c-Myc signal. So if DNAase is treated, it will abrogate the entry of DNA and subsequent activation of the pathway. Hence, the results are not conclusive to show that DNA transferred from the tumor to the recipient cell has lead to the expression of the proteins. Either this sentence has to be omitted or the experiment has to be repeated with human specific c-Myc.
