# Supplementary material for: Therapeutic interventions on human breast cancer xenografts promote systemic dissemination of oncogenes
Source: PLoS One. 2024 Feb 12;19(2):e0298042. doi: 10.1371/journal.pone.0298042 (PMC10861051; doi:10.1371/journal.pone.0298042)
Supplement: Attachment — Submitted filename: Response to Reviewers and Editor.docx [file pone.0298042.s008.docx]

Dear Dr. Vijayakurup

We have complied with comment no. 1 of reviewer 2 wherein a suggestion was made that we perform an experiment in which we inject fluorescently labelled MDA-MB-231 cells to prove that the fluorescent signals that we observed in brain cells of mice were not metastatic cells.

We have now performed such an experiment which is described in the “Methods” section; the results of which are described in the “Results” section, and the conclusions discussed in the “Discussion” section. This has required substantial changes to the manuscript (marked in red) and incorporation of a new figure marked as “Supplementary Fig. 3”.

5. Review Comments to the Author

Please use the space provided to explain your answers to the questions above. You may also include additional comments for the author, including concerns about dual publication, research ethics, or publication ethics. (Please upload your review as an attachment if it exceeds 20,000 characters)

Reviewer #1: The manuscript “Therapeutic interventions on human breast cancer xenografts promote systemic dissemination of oncogenes” is a small piece of work that explains probable reason for tumor relapse or metastasis after successfully treating the tumor with commonly adopted therapeutic modalities like chemotherapy, radiotherapy and surgery.

The authors hypothesize that therapeutic interventions lead to the dispersal of DNA fragments from the dying cancer cells, which carrying the oncoprotein. This is transported by blood to distant organ sites to induce tumor development and the use of agents that can deactivate these cell free DNA particles can lead to therapeutic benifits.

Authors have shown by immuno-FISH analysis, in mice xenograft brain sections, the co-localization of human DNA with HLA-ABC antigen, which is specific to human and different human oncogenes, after therapeutic interventions. The group has also suggested and shown that use of cell free chromatin particles deactivating agents along with the different therapies could inactive and prevent these cell free chromatin particles from inducing the toxic side effects.

One point I want to bring to notice is that the figures are blurred. Please enhance the figure quality.

The quality of the figures has now been enhanced. Thank you.

Reviewer #2: The manuscript titled “Therapeutic interventions on human breast cancer xenografts promote systemic dissemination of oncogenes” describes a potential transfer of cell-free chromatin particles from tumor cells to normal cells in response to different treatment modalities. The novelty of the study is overall low and the experimental evidences for the interpretations made are weak.

Some of the points to be addressed are given below

Major points

1. The results do not explicitly show that the signals detected in brain sections are indeed from the cfChPs released from the xenograft. It can be caused due to metastasis of the tumor to brain. Ideally, MDA-MB-231 cells should have been labelled with a fluorescent protein to detect its presence in the brain. An absence of this signal with the presence of the human DNA sequences will confirm the transfer of cfChPs from the tumor to the recipient cells.

We thank the reviewer for making this valuable suggestion. We have now performed the experiment as suggested wherein we have dually fluorescently labeled MDA-MB-231 cells in their DNA with BrdU and in their histones with H2B-GFP by a method described by us earlier (reference no. 16 of the manuscript). The florescent cells were injected i.v. and brain cells were examined after 72 h. No intact fluorescent cells representing metastasis were seen in the brain. What we saw were dually labelled particle representing cfChPs. This confirms our hypothesis that cfChPs from dying xenograft cells go and accumulate in the brain cells and express the onco-proteins that they carry with them.

This data has now been incorporated as Supplementary fig. 3 in the manuscript.

The above suggestion of the reviewer and the experimental procedure has required the addition of the following paragraphs:

1) pp. 10, lines 163-176 (Methods section)
2) pp. 12, lines 222-231 (Results section)
3) pp. 13, lines 239-249 (Discussion section)
4) pp. 13-14, lines 251-258 (Discussion section)

This has also necessitated reorganizing the references.

2. As a first step, the investigators could have assessed human circulating cell-free DNA in response to different treatment modalities compared to control (Xenograft only) and see if it correlates with a higher signal in the brain.

We humbly submit that in light of our response to reviewer’s point no. 1 above, this experiment is no longer required.

3. Another approach to confirm the transfer of cfChPs to the host recipient cell is by blocking endocytosis and see if there is reduction in the immuno-FISH signal. This can be first tested in vitro. ALternatively, try an approach similar to the one used in Mittra et al.,Cell Death and Discovery.2017(referred in the manuscript).

We humbly submit that in light of our response to reviewer’s point no.1 above, this experiment is no longer required.

4. What is the rationale for choosing the proteins (p-EGFR,p-Akt,c-Myc,Raf,PDGFRA,HRAS,FGFR) listed in the results?

There was no specific rationale except that these antibodies were readily available in our laboratory. The important point is that they are all onco-proteins.

5. The results are not conclusive to show that DNA transferred from the tumor to the recipient cell has lead to the expression of the proteins (first paragraph of results). It could also be due to transfer of these proteins from the tumor cells via other routes like extracellular vesicles. Hence, this interpretation should be avoided.

Had the transfer of the onco-proteins from the tumor cells had occurred via other routes, such as via extracellular vesicles, they would not have been abrogated by treatment with the three cfChPs deactivating agents.

Minor points

1. NF-ƘB is a transcription factor and not a cytokine. Correct this in the introduction.

We thank the reviewer for pointing this out. NFκB has now been appropriately specified (pp. 3, line 55).

2. Typing error in materials and methods under the heading "creation of xenografts"- MDA-MB-213 written instead of 231

Sorry for this typo. This has now been corrected (pp. 5, line 104).
