# Supplementary material for: Therapeutic interventions on human breast cancer xenografts promote systemic dissemination of oncogenes
Source: PLoS One. 2024 Feb 12;19(2):e0298042. doi: 10.1371/journal.pone.0298042 (PMC10861051; doi:10.1371/journal.pone.0298042)
Supplement: Attachment — Submitted filename: Rebuttal letter.docx [file pone.0298042.s010.docx]

Review Comments to the Author

Please use the space provided to explain your answers to the questions above. You may also include additional comments for the author, including concerns about dual publication, research ethics, or publication ethics. (Please upload your review as an attachment if it exceeds 20,000 characters)

Reviewer #1: The Figures are still not to the quality of publication I feel. Not much change has been made into the figure quality then the previous version of the manuscript.

We are once again providing a high resolution images of all the figures and supplementary figures. We have used DPI Converter using the following link. All images have been enhanced to 600 dpi.

<https://convert.town/image-dpi>

Reviewer #2: 1. Supplementary figure.3 has to be replaced by a more high resolution image.
This question has been answered above

2. A query related to Point no.5 from the previous review- In the manuscript, the authors have shown increased c-Myc signal in the brain of tumor bearing animals with/without therapeutic intervention at varying intensity compared to control group where there is no signal. However, the antibody used here is reactive to mouse c-Myc and human c-Myc. Hence, the question of specificity still remains.

We like to point out that we have used the Sigma Aldrich anti-human c-Myc monoclonal antibody M4439 which is clearly indicated in supplementary table 1.

This mAb is human specific as indicated in the company datasheet which is given as “supporting information”. The datasheet contains the following specificity statements:

Species reactivity: Human

Immunogen: Synthetic peptide of the human p62c-Myc protein.

Moreover, the possibility that the internalized DNA could be activating pathways like cGAS-STING cannot be ruled out. These pathways could be responsible for the elevated c-Myc signal. So if DNAase is treated, it will abrogate the entry of DNA and subsequent activation of the pathway. Hence, the results are not conclusive to show that DNA transferred from the tumor to the recipient cell has lead to the expression of the proteins. Either this sentence has to be omitted or the experiment has to be repeated with human specific c-Myc.

Since the mAb is human specific, the possibility that cGAS-STING pathway is responsible for the elevated c-Myc signal is ruled out.

A rebuttal letter that responds to each point raised by the academic editor and reviewer(s). You should upload this letter as a separate file labeled 'Response to Reviewers'.

A marked-up copy of your manuscript that highlights changes made to the original version. You should upload this as a separate file labeled 'Revised Manuscript with Track Changes'.

An unmarked version of your revised paper without tracked changes. You should upload this as a separate file labeled 'Manuscript'.
