# Supplementary material for: Therapeutic interventions on human breast cancer xenografts promote systemic dissemination of oncogenes
Source: PLoS One. 2024 Feb 12;19(2):e0298042. doi: 10.1371/journal.pone.0298042 (PMC10861051; doi:10.1371/journal.pone.0298042)
Supplement: Attachment — Submitted filename: revision 3.docx [file pone.0298042.s011.docx]

The authors have answered most of the queries raised by the reviewers. However, I have noticed that in Figure 1b shows localization of PDGFRA, HRAS, c-Myc, c-Raf etc in the nucleus. While there are reports of transfer of membrane proteins to the nucleus, an exclusive localization to the nucleus is not common. Moreover, the significance of the result is not very clear. What is the relevance of localization of the chromatin particles with the mentioned oncogenic proteins in the nucleus? Please mention this in the manuscript.
