# Supplementary material for: Therapeutic interventions on human breast cancer xenografts promote systemic dissemination of oncogenes
Source: PLoS One. 2024 Feb 12;19(2):e0298042. doi: 10.1371/journal.pone.0298042 (PMC10861051; doi:10.1371/journal.pone.0298042)
Supplement: Attachment — Submitted filename: 1. Response to Reviewers.docx [file pone.0298042.s012.docx]

**Response to Reviewers**

Reviewer #2: The authors have answered most of the queries raised by the reviewers. However, Figure 1b shows localization of PDGFRA, HRAS, c-Myc, c-Raf etc in the nucleus. While there are reports of transfer of membrane proteins to the nucleus, an exclusive localization to the nucleus is not common. Moreover, the significance of the result is not very clear. What is the relevance of localization of the chromatin particles with the mentioned oncogenic proteins in the nucleus? Please mention this in the manuscript.

We agree with the reviewer that there are several reports in the literature of transfer of membrane proteins to the nucleus (for example, Zuleger, N., et al. 2012. Cell. Mol. Life Sci., 69, 2205-2216 and Zheng, H.C. et al. 2022. Mol. Med. Reports, 25, 1-12.). However, in our case, the oncogenes contained within the cfChPs that had accumulated in brain cells were being directly expressed as proteins in their nuclei. The relevance of such a finding is that oncogenes carried by cfChPs via the blood stream to the brain can potentially transform the brain cells to form new cancers which would masquerade as metastasis.

We have now addressed this issue on page 14, lines 282-287

Reviewer #3: Major concerns:

• Data presented is preliminary and requires extensive validation. For example, the data obtained by immuno-fluorescence and FISH analysis are only suggestive, further work is still needed to establish that it is cell free oncogenic DNA and proteins rather than MDA-MB-231 cells.

This issue was also raised earlier by the second reviewer who had suggested that we conduct the experiment which is now presented in supplementary figure 3. The latter shows that no intact intravenously injected MDA-MB-231 cells are present in the brain. The second reviewer is now satisfied with this experiment.

We believe that the experiment described in Supplementary Figure 3 conclusively excludes the possibility that the fluorescence signals seen in Figure 1 represent intact MDA-MB-231. In this experiment we intravenously injected dually fluorescently labelled MDA-MB-231 cells and looked for intact fluorescent cells in the brain of mice. Intact cells would be expected to appear as large dually-labelled fluorescent signals which would co-localize with an entire DAPI nuclear signal. We did not find any such large co-localizing signals; instead we detected fluorescent particles representing cfChPs within the blue DAPI nuclear signals. This suggested that cfChPs from dying xenograft cells had been carried via the blood stream to accumulate in the brain cell nuclei.

Additionally, the fact that the fluorescent signals could be markedly minimized by concurrent treatment with the three cfChPs deactivating enzymes strongly argues against the possibility that the fluorescent signals seen in brain cells represented intact MDA-MB-231 cells. Rather, they indicate that the vehicles that carried the c-Myc oncogene to brain cells were cfChPs released from dying xenograft cells which could be prevented by deactivating cfChPs.

We have now addressed the reviewer’s concerns in two separate paragraphs in the discussion section (pages 13-14, lines 259-281).

One way to address this issue is by immunohistochemistry, which was not done.

The reviewer does not specify which antigen should be used as target for immunohistochemistry. After extensive search, we could not find a surface antigen which is unique to MDA-MB-231 cells and is not present in brain cells. For example, the epithelial cell surface marker ICAM-1 is also expressed by microglial cells in the brain.

Moreover, the work described in supp. Figure 3 is difficult to interpret considering the difference in timeframe between the original work (figures 1 and 2), which lasted for 6 weeks and the work in supp. Figure 3, which lasted for only 72 hours.

We agree that there is difference in the timeframe between figures 1 & 2 and supplementary figure 3. However, if no intact MDA-MB-231 cells in the brain were seen at 72 h (Supplementary Fig. 3), it is highly unlikely that the fluorescent signals seen at 6 weeks would represent intact MDA-MB-231 cells (Figures 1 and 2). In fact, the absence of intact cells at 72 h is in keeping with Fidler’s finding that 99% of injected cancer cells rapidly die within 24 h [11].

We have now addressed the issue of difference in timeframe in the discussion section (Page 14, Lines 267-273)

• Using a xenograft mouse model to address this issue is problematic for many reasons, not least of which is that these are loose cells that can travel to different organs. A more suitable model is to work with a model of chemically- or virally-induce cancer. This way, the investigation starts with a localized cancer and oncogenic release of cell free DNA or proteins, rather than intact cells, can be tracked with greater confidence.

We beg to disagree. The induction of chemically- or virally-injected tumours locally would represent an unnatural scenario and not represent the real world of human cancer (such as breast cancer).

• The study is devoid of any mechanistic insight; experimentally speaking, how can cell free DNA and/or protein promote metastasis as per the model used in this study.

The mechanism that underpins our findings was described by us earlier wherein we had shown that cfChPs that circulate in blood of cancer patients can readily enter into cells of distant organs to damage their DNA and potentially transform them into new cancers which could appear as metastasis (Mittra, I., et al. (2015). J. biosci., 40, 91-111).

We have now addressed this mechanistic aspect of our finding in the manuscript (Page 13, Lines 255-258)

• There is a serious logical disconnect between the hypothesis put forward by the authors, which says that “therapeutic interventions may disseminate the disease 31 via medium of cfChPs released from therapy induced dying cancer cells” and the some of the validation work (results lines 219-228 & supp. Figure 3) that was done to rule out that the signals detected in mouse brains were not metastatic cells?

We thank the reviewer for pointing out this rather confusing interpretation of the hypothesis on our part. This is indeed a very important issue that requires detailed clarification.

The clarification that fluorescent signals seen in brain cells were cfChPs and not intact cells has been addressed above. Further clarification that cfChPs, rather than intact cells, are the potential agents that can transform target cells to generate new cancers that would masquerade as metastasis required clarification at several places

These clarifications have been made in several sentences in the abstract (marked in red); in several places in the introduction section (marked in red). The discussion section has been extensively re-written to emphasize this novel mechanism of metastasis which poses a formidable challenge to the existing model (marked in red).

Minor comments:
• There are no legends for figures 1 and 2

There was a lot of confusion in the editorial office after we uploaded this manuscript, and were subsequently asked to send the manuscript by email. We have confirmed from our folder that we had sent the legends to figures as a separate file in our email to the editorial office. There seems to have been some error on part of the editorial office because of which the legends are missing. For the benefit of the reviewer we are reproducing below legends to figures 1 & 2.

Legends to figures

Fig.1: Representative immuno-FISH images of FFPE sections of brains of mice bearing human breast cancer xenografts (without therapeutic interventions) showing co-localizing signals of human DNA and various human onco-proteins. a. Co-localizing signals of human DNA and human HLA-ABC. b. Co-localizing signals of human DNA and eight different human onco-proteins.

Fig. 2: Quantitative analyses depicted as histograms to illustrate that therapeutic interventions promote systemic dissemination of human DNA and human c-Myc onco-protein to mouse brain cells, and that these can be prevented by concurrent treatment with three different cfChPs deactivating agents. All groups had 4 mice each. a. Detection of human DNA by FISH b. Detection of human c-Myc onco-protein by immunofluorescence. Statistical comparison between the xenograft bearing group and the two control groups, and that between the xenograft bearing group and the three anti-cancer treatment groups (CT, RT and Sx) was done by two-tailed student t-test. * < 0.05, ** < 0.01, *** <0.001, **** <0.0001. Statistical comparison between the three anti-cancer treatment groups (CT, RT and Sx) and those additionally treated with the three cfChPs deactivating agents was done by One-way ANOVA. * < 0.05, ** < 0.01.

• The introduction is almost a repeat of the abstract

We thank the reviewer for pointing this out. We have now re-written the introduction to give it a different connotation (marked in red).

• The discussion is very short and barely addresses the findings in the context of what is already known regarding this phenomenon

We thank the reviewer for making this observation. The discussion section has now been extensively re-written and enlarged (marked in red).
