# Supplementary material for: Therapeutic interventions on human breast cancer xenografts promote systemic dissemination of oncogenes
Source: PLoS One. 2024 Feb 12;19(2):e0298042. doi: 10.1371/journal.pone.0298042 (PMC10861051; doi:10.1371/journal.pone.0298042)
Supplement: Attachment — Submitted filename: 1. Response to Reviewers.docx [file pone.0298042.s013.docx]

**Revision 3**

**This question actually pertains to review no. 2 to which we had already responded.**

3. The authors have answered most of the queries raised by the reviewers. However, I have noticed that in Figure 1b shows localization of PDGFRA, HRAS, c-Myc, c-Raf etc in the nucleus. While there are reports of transfer of membrane proteins to the nucleus, an exclusive localization to the nucleus is not common. Moreover, the significance of the result is not very clear. What is the relevance of localization of the chromatin particles with the mentioned oncogenic proteins in the nucleus? Please mention this in the manuscript.

We agree with the reviewer that there are several reports in the literature of transfer of membrane proteins to the nucleus (for example, Zuleger, N., et al. 2012. Cell. Mol. Life Sci., 69, 2205-2216 and Zheng, H.C. et al. 2022. Mol. Med. Reports, 25, 1-12.). However, in our case, the oncogenes contained within the cfChPs that had accumulated in brain cells were being directly expressed as proteins in their nuclei. The relevance of such a finding is that oncogenes carried by cfChPs via the blood stream to the brain can potentially transform the brain cells to form new cancers which would masquerade as metastasis.

We have now addressed this issue on page 14, lines 282-287

**Revision 2**

**The below question is the only new question this time round**

1. Supplementary figure.3 has to be replaced by a more high resolution image.

We have already provided high resolution image of Supplementary Figure 3. We used the DPI Converter using the following link. All images have been enhanced to 600 dpi.

<https://convert.town/image-dpi>

Perhaps the reviewer has been provided with a PDF version of the image.

Nonetheless, we are again uploading the same high resolution image.

**The below question actually pertains to review no. 1 to which we had already responded.**

2. Point no.5 from the previous review (reviewer 2) has not been addressed properly. In the manuscript, the authors have shown increased c-Myc signal in the brain of tumor bearing animals with/without therapeutic intervention at varying intensity compared to control group where there is no signal. However, the antibody used here is reactive to mouse c-Myc and human c-Myc. Hence, the question of specificity still remains.

We like to point out that we have used the Sigma Aldrich anti-human c-Myc monoclonal antibody M4439 which is clearly indicated in supplementary table 1.

This mAb is human specific as indicated in the company datasheet which is given as “supporting information”. The datasheet contains the following specificity statements:

Species reactivity: Human

Immunogen: Synthetic peptide of the human p62c-Myc protein.

Moreover, the possibility that the internalized DNA could be activating pathways like cGAS-STING cannot be ruled out. These pathways could be responsible for the elevated c-Myc signal. So if DNAase is treated, it will abrogate the entry of DNA and subsequent activation of the pathway. Hence, the results are not conclusive to show that DNA transferred from the tumor to the recipient cell has lead to the expression of the proteins. Either this sentence has to be omitted or the experiment has to be repeated with human specific c-Myc.

Since the mAb is human specific, the possibility that cGAS-STING pathway is responsible for the elevated c-Myc signal is ruled out.
