# Supplementary figures and images for: Correction: Therapeutic interventions on human breast cancer xenografts promote systemic dissemination of oncogenes
Source: PLoS One. 2024 Mar 28;19(3):e0301650. doi: 10.1371/journal.pone.0301650 (PMC10977690; doi:10.1371/journal.pone.0301650)

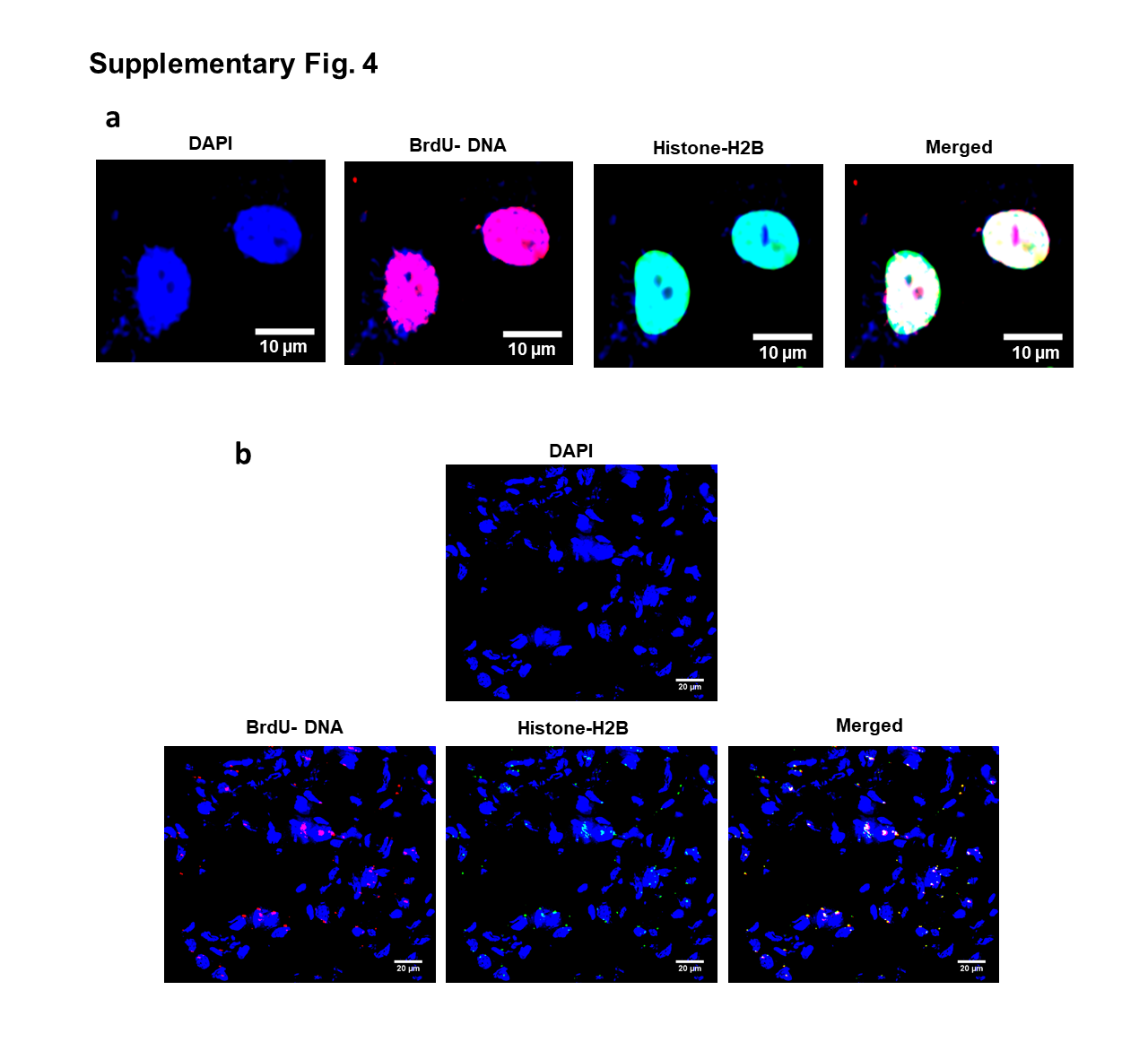

Supplement: S4 Fig — Figure showing fluorescently dual-labelled cfChPs in mouse brain (a and b): Fluorescently dually labelled MDA-MB-231 cells when injected intravenously into SCID mice die upon reaching the brain and release cfChPs which accumulate in their brain cells. a. Representative image of dually fluorescently labelled MDA-MB-231 cells. b. Fluorescent microscopy image of brains of mice injected with dually labelled MDA-MB-231 cells to demonstrate that the cells die upon reaching the brain to release dually labelled fluorescent particles representing cfChPs which accumulate in their brain cells. (TIF) [file pone.0301650.s001.tif]
